# A salmon diet database for the North Pacific Ocean

**DOI:** 10.1038/s41597-020-00676-y

**Published:** 2020-10-06

**Authors:** Caroline Graham, Evgeny A. Pakhomov, Brian P. V. Hunt

**Affiliations:** 1 Institute for the Oceans and Fisheries, University of British Columbia, 2202 Main Mall, Vancouver, British Columbia V6T 1Z4 Canada; 2 Department of Earth, Ocean, and Atmospheric Sciences, University of British Columbia, 2207 Main Mall, Vancouver, British Columbia V6T 1Z4 Canada; 3 Hakai Institute, PO Box 25039, Campbell River, British Columbia V9W 0B7 Canada

**Keywords:** Marine biology, Food webs, Ichthyology

## Abstract

The North Pacific Marine Salmon Diet Database is an open-access relational database built to centralize and make accessible salmon diet data through a standardized database structure. The initial data contribution contains 21,862 observations of salmon diet, and associated salmon biological parameters, prey biological parameters, and environmental data from the North Pacific Ocean. The data come from 907 unique spatial areas and mostly fall within two time periods, 1959–1969 and 1987–1997, during which there are more data available compared to other time periods. Data were extracted from 62 sources identified through a systematic literature review, targeting peer-reviewed and gray literature. The purpose of this database is to consolidate data into a common format to address gaps in our ecological understanding of the North Pacific Ocean, particularly with respect to salmon. This database can be used to address a variety of questions regarding salmon foraging, productivity, and marine survival. The North Pacific Marine Salmon Diet Database will continue to grow in the future as more data are digitized and become available.

## Abstract (summary)

**Table:** 

Measurement(s)	Gastric Content • diet
Technology Type(s)	digital curation • microscopy
Factor Type(s)	predator capture method • year of data collection • longitude • latitude • predator life stage • predator lowest taxonomic level • predator characteristics • predator replicates • type of diet data • prey life stage • prey lowest taxonomic level • prey characteristics
Sample Characteristic - Organism	Oncorhynchus mykiss • Oncorhynchus kisutch • Oncorhynchus tshawytscha • Oncorhynchus nerka • Oncorhynchus keta • Oncorhynchus gorbuscha
Sample Characteristic - Environment	ocean
Sample Characteristic - Location	North Pacific Ocean

Machine-accessible metadata file describing the reported data: 10.6084/m9.figshare.12981989

## Background & Summary

Even though salmon spend 1–6 years of their life in the marine environment, this phase of their life cycle is poorly understood compared to their freshwater phase^1^. There are limited data on how salmon are distributed, what they feed on, and what threats to survival they may face during this phase, which includes nearshore and offshore components. The marine phase is hypothesized to contain salmon population bottlenecks^2^, and research has shown that salmon smolt to adult survival rates can be less than 1% for some stocks in the North Pacific^3^. There is also evidence that Pacific salmon marine survival has been declining over the past several decades in certain areas, especially more southern stocks^4,5^. Therefore, it is becoming urgent that researchers understand what is happening to salmon during the marine phase of their life cycle, especially after they have moved offshore, because this phase tends to be data-poor compared to the early marine coastal phase.

One of the most important factors affecting salmon survival is the presence and abundance of suitable prey. Although it is difficult to assess prey distribution across ocean basins, information on prey presence and abundance can be derived from salmon diets. Marine diet data has the potential to give insight into food webs, niche overlap among species/stocks, competition, health, and changing ocean conditions^6–8^. Since the early 1900s, researchers have been examining the diets of Pacific salmon to provide information on species biology and the conditions that salmon face in the ocean^9,10^. Although there have been some reviews of salmon diet data in the North Pacific, these have been limited in time and space and the data are not normally made publicly available^7,8,11–15^.

Salmon diet data have been collected using a variety of methodologies employed by researchers from countries across the North Pacific. These data are scattered across the peer-reviewed and gray literature, making collation challenging. However, there is high value in collating these data, considering the costliness and difficulty of conducting fieldwork in the open ocean. Synthesizing these data can reveal important information about salmon open ocean life history experience and further understanding of the potential impacts of changing oceans on salmon productivity. Additionally, more comprehensive, accurate and robust diet data will be an asset to ecosystem models, which are increasingly being applied in ecosystem-based management^16^. As salmon face an uncertain future with climate change^17,18^, this is a critical time to consolidate available knowledge in order to advance research on salmon marine ecology.

The goal of this project was to develop an open-access database framework for collating marine salmon diet data, alongside available salmon biological data, prey biological data, and environmental data. We compiled an initial contribution of salmon stomach content diet data from offshore areas using a systematic literature review, followed by quality control and standardization procedures for two time periods: 1959–1969 and 1987–1997. These decades were selected partially because they are time periods in which there are a larger quantity of data available on salmon diets. This database will continue to grow as more sources are identified and added and can be used as a tool by researchers to study salmon marine survival and North Pacific ecosystem dynamics.

## Methods

### Systematic literature review

In order to identify sources that contained salmon diet data, in the form of stomach contents, a systematic literature review was performed using database keyword searches of ProQuest: Aquatic Sciences and Fisheries Abstracts (https://search.proquest.com/asfa), Web of Science: Core Collection and Web of Science: Zoological Record (https://webofknowledge.com). Not all salmon diet studies are part of the peer-reviewed literature and many North Pacific researchers have published data through the North Pacific Anadromous Fish Commission (NPAFC) and the defunct International North Pacific Fisheries Commission (INPFC). Therefore, database search results were supplemented with relevant INPFC documents and bulletins (https://npafc.org/inpfc/), NPAFC documents (https://npafc.org/npafc-documents/) and bulletins (https://npafc.org/bulletin/), and relevant bibliographic references found within these documents and bulletins (Table 1).

**Table 1:** The number of salmon diet data sources identified from a systematic literature review.

Search terms	Source	Results before filtering	Results after filtering
(Chinook OR “Oncorhynchus tshawytscha” OR coho OR “Oncorhynchus kisutch” OR sockeye OR “Oncorhynchus nerka” OR pink OR “Oncorhynchus gorbuscha” OR chum OR “Oncorhynchus keta” OR steelhead OR “Oncorhynchus mykiss”) AND (marine OR ocean* OR coast* OR “Gulf of Alaska” OR “Bering Sea”) AND (stomach OR gut* OR “prey composition” OR “diet* composition” OR “composition of diet” OR “composition of prey”) AND (diet* OR prey OR food)	Proquest: Aquatic Sciences and Fisheries Abstracts	410	23
	Web of Science: Core Collection	182	6
	Web of Science: Zoological Record	142	12
	North Pacific Anadromous Fish Commission Documents		23
	North Pacific Anadromous Fish Commission Bulletins		16
	International North Pacific Fish Commission Documents		5
	International North Pacific Fish Commission Bulletins		6
			Total = 62

A keyword search was used to identify sources in three online databases (Proquest: Aquatic Sciences and Fisheries Abstracts, Web of Science: Core Collection, Web of Science: Zoological Record), which contained most of the peer-reviewed literature. The former steelhead species name “*Salmo gairdneri*”, when included as a search term, did not provide any more relevant sources that met our criteria. A manual search through the NPAFC and INPFC documents and bulletins provided most of the gray literature. A total of 62 unique sources met the qualifications for database entry.

The database keyword searches identified 591 unique sources. Sources were filtered for relevance and excluded based on the following criteria:

(i) the source did not have salmon stomach content diet data from between 1959–1969 or 1987–1997 for the marine environment, as defined by the area beyond the Riverine Coastal Domain (~15 km)^19^ (556 sources);
(ii) the source did not have extractable diet data and authors did not respond to inquiries (1 source);
(iii) the source was a review, in which case the original sources were used, if possible and relevant, to extract data (2 sources);
(iv) the source overlapped completely with another source, i.e., the data were reported using the exact same metrics for the same samples as another source (2 sources).

The database search was supplemented with sources that met the same criteria from the INPFC documents and bulletins and the NPAFC documents and bulletins, bringing the total number of unique sources to 62 (Online-only Table 1).

### Data extraction

For each salmon diet sample, we extracted the following data (if available): source metadata (e.g., publication year, title, authors) (Online-only Table 2), salmon capture method (Online-only Table 3), site information (time, location), salmon information (e.g., taxonomy, life stage, sex), salmon replicates, type of diet data (e.g., percent weight of prey, total number of prey), and prey information (e.g., taxonomy, life stage, quantity) (Online-only Table 4). A diet sample is defined as a distinct sample in time and space from a specific source and is entered into the database exactly as it is reported in the source. A sample can contain diet data from one salmon or more than one salmon when individuals were grouped together for diet analysis (up to 2,215 in this data compilation). If different diet metrics were reported for the same diet sample (e.g., number of prey and volume of prey), then all metrics were entered into the database. If the data were not available in table format, but figure format only, then the data were extracted using WebPlotDigitizer (https://apps.automeris.io/wpd/).

For each source, we extracted data as it was presented in the sources in almost all cases, and therefore it was extracted according to the data resolution of the source. For example, if the sample location was presented as a station, it was extracted as a station with specific geographical coordinates, but if it was presented as a transect or area, then it was extracted as a transect or area with latitude and/or longitude minimums and maximums. If geographical coordinates were not specified, then they were estimated based on survey maps or descriptions present in the source. Prey taxonomy was reported to different resolutions across sources (e.g., Copepoda versus *Neocalanus cristatus*). In order to keep the lowest data resolution reported in the source while also being able to compare across sources, each prey item was reported at all possible taxonomic levels (kingdom, phylum, class, order, family, genus, species). In addition to the salmon diet data, if the source presented additional related data for salmon biological parameters (i.e., variables) (Online-only Table 5), prey biological parameters (Online-only Tables 6 and 7), or environmental parameters (Online-only Table 8), these data were extracted as well. For detailed information about the different types of data extracted and the extraction methodology see Online-only Tables 2–8.

### Database framework

We built an open-access relational database in MySQL v8.0.18 called the “North Pacific Marine Salmon Diet Database”^20,21^. The North Pacific Marine Salmon Diet Database contains all of the extracted data noted above: diet data, salmon biological data, prey biological data, and environmental data. This database also allows for inclusion of prey biological data that are not associated with a salmon sample. For example, if a researcher conducted a zooplankton tow for potential prey and they have biological data for these potential prey (e.g., length, weight), these data can be added to the database. In this database, all data are linked by site, which has both a temporal and spatial component. All data are also related to a source in order to distinguish related data and trace its origins. While the database was built specifically to house North Pacific salmon diet data from the marine environment, the database structure was designed to easily be applied to other predator and prey interactions with only slight modifications.

## Data Records

The North Pacific Marine Salmon Diet Database currently contains 6,869 diet observations from 6,305 unique diet samples of over 69,942 salmon. Types of diet data included percent weight of prey, absolute weight of prey, average weight of prey, percent volume of prey, percent number of prey, absolute number of prey, average number of prey, frequency of occurrence (numerical and percent), stomach content index, index of fullness, and index of relative importance. The database also houses 11,965 observations of salmon biological parameters for 6,172 unique salmon samples. One observation means one biological parameter measured for one sample of salmon, which can contain one or many fish. Salmon biological parameters include length, weight, daily ration, empty stomachs, male/female ratio and many others. Additionally, the database includes 238 observations of prey biological parameters from 112 unique prey taxonomic categories. Prey biological parameters include body length, body weight, body width and size index. Finally, the database contains 2,790 observations of environmental parameters. Environmental parameters include temperature, salinity and others. These data are available in a static figshare repository^22^. The most current version of the relational database, associated documentation, and data are available in a dynamic GitHub repository, which will be updated as more sources are identified and added to the database^21^. Data were visualized using R statistical software v3.6.1^23^.

### Spatial and temporal coverage

**Fig. 1:**
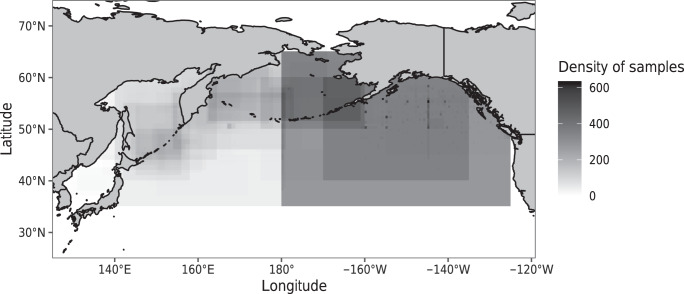
The spatial distribution of diet samples across the North Pacific Ocean. The density of diet samples, in the form of points, lines, and polygons (rectangles) based on the latitude and longitude minimum and maximum values.

**Fig. 2:**
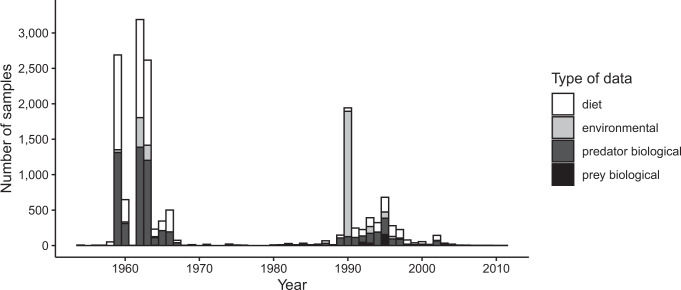
The number of samples for each type of data reported across the temporal range 1950–2011. If a single sample consisted of data from multiple years, then the median year was selected to represent the sample and half years were rounded down.

Diet samples were collected at 751 unique spatial locations, which included areas (polygons), transects, and point locations across the North Pacific from the California Current to the Sea of Japan (Fig. 1). Salmon biological data were collected from 709 locations, prey biological data from 4 locations, and environmental data from 446 locations. Salmon biological data were reported across the entire North Pacific, while prey biological data were sparsely reported from a few locations in the Gulf of Alaska and the Sea of Okhotsk/Kuril Islands. Environmental data were mainly available from the eastern and central North Pacific Ocean. Since our search was focused on specific time periods, most of the data we collected fell within our specified decadal periods: 1959–1969 and 1987–1997 (Fig. 2). However, some sources reported data from other time periods and these data were also included in the database. While diet data and salmon biological data were consistently reported across the temporal range, environmental data and prey biological data were inconsistently reported.

### Salmon and prey species coverage

Sockeye (*Oncorhynchus nerka*), pink (*Oncorhynchus gorbuscha*) and chum (*Oncorhynchus keta*) were reported most frequently in our database, while coho (*Oncorhynchus kisutch*), Chinook (*Oncorhynchus tshawytscha*) and steelhead (*Oncorhynchus mykiss*) were reported less frequently (Table 2). The most commonly reported prey groups were amphipods, fish (Class Actinopterygii), euphausiids, cephalopods (Subclass Coleoidea), and copepods (Table 3). The category ‘miscellaneous’ was also commonly reported, although it usually made up just a small percentage of the diets. Within the diet data reported in the database, there are 186 unique prey taxa, meaning the lowest taxonomic classifications of prey items. Only 18.5% of salmon diet data were reported to the species level, while the majority were reported to higher taxonomic levels (e.g., Amphipoda, Decapoda, Euphausiacea).

**Table 2:** The number of diet samples in the database for each species of salmon.

Salmon species	Number of samples
Sockeye (*Oncorhynchus nerka*)	2287
Pink (*Oncorhynchus gorbuscha*)	1768
Chum (*Oncorhynchus keta*)	1526
Coho (*Oncorhynchus kisutch*)	293
Chinook (*Oncorhynchus tshawytscha*)	271
Steelhead (*Oncorhynchus mykiss*)	160

**Table 3:** The number of diet samples containing each prey taxonomy.

Prey taxonomy	Number of samples
Amphipoda	2446
Actinopterygii	2436
Euphausiacea	2153
Miscellaneous	2026
Coleoidea	2009
Copepoda	1460
Pteropoda	697
Limacina	595
*Themisto japonica*	584
*Thysanoessa longipes*	525
*Limacina helicina*	438
Brachyura	389
*Neocalanus cristatus*	353
*Clione limacina*	319
Hyperiidae	299
Hemilepidotus	290
Decapoda	241
Anomura	200
Gelatinous collective	195
*Euphausia pacifica*	180
Myctophidae	179
*Hyperia medusarum*	148
Polychaeta	140
Calanoida	131
Chaetognatha	122
Cephalopoda	118
*Parasagitta elegans*	115
*Primno macropa*	110
Sagitta	96
Euphausiidae	90
*Rhynchonereella angelini*	79
*Eucalanus bungii*	74
*Neocalanus plumchrus*	73
Insecta	68
*Thysanoessa raschii*	68
Oikopleura	67
*Gadus chalcogrammus*	60
*Clupea pallasii*	56
Gammaridae	55
*Themisto pacifica*	55
*Gonatopsis borealis*	49
Crustacea	47
*Hyperia galba*	44
*Mallotus villosus*	43
*Gonatus kamtschaticus*	40
Mysida	40
*Ammodytes hexapterus*	39
Ammodytidae	35
*Thysanoessa inspinata*	35
Gonatidae	33
Hexagrammidae	32
Cnidaria	29
Pleurogrammus	28
Harpacticoida	27
Themisto	27
*Berryteuthis magister*	26
*Engraulis japonicus*	26
Nekton collective	26
*Thysanoessa inermis*	26
Tunicata	25
Appendicularia	23
*Leuroglossus schmidti*	22

Prey taxonomy refers to the lowest taxonomic level identified by the source. Prey taxonomies that were reported in less than 20 samples were excluded.

## Technical Validation

Standardization procedures were used to verify and collate the data. Since some of the taxonomic records were outdated, the taxonomies were verified and updated using the World Register of Marine Species (http://www.marinespecies.org/). For types of diet data that should add to a cumulative percentage of 100 (e.g., percent weight, percent volume), diet samples were excluded if the cumulative percent of prey was above 105% or below 95%. If the cumulative percentage did not add to 100 but still fell within this range, diet data values for that sample were rescaled to add to 100. For other metrics, including absolute and average weight and number of prey, as well as numerical frequency of occurrence, we consulted a salmon diet expert to determine if our highest values were reasonable to find in adult salmon stomachs (V. Zahner, pers. comm.).

## Usage Notes

The North Pacific Marine Salmon Diet Database contains observations from many different sources, which report data with varying levels of detail. The method for data digitalization was to extract data as it was presented in the sources, or as close to it as possible. In this way, we have provided a more complete set of data that can be used for a variety of studies on salmon ecology and North Pacific ecosystems and left the manipulation and interpretation of data up to the discretion of the user.

The North Pacific Marine Salmon Diet Database is publicly available and can be used under the license of CC BY, meaning that the work can be distributed, remixed, adapted and built upon with acknowledgement of authors. There are a number of ways to access the information contained in the database. A static copy of the initial data contribution to the North Pacific Marine Salmon Diet Database described in this paper is available in a figshare repository in the form of seven csv files (‘Diet_data’, ‘Prey_biological_data’, ‘Sources’, ‘Predator_biological_data’, ‘Environmental_data’, ‘Gear_type_prey’, ‘Gear_type_predator’)^22^. In this case, ‘predator’ refers to salmon, but the database was designed so that it could easily be applied to other predator and prey interactions and thus we used the predator-prey terminology. Detailed information about the variables in the database can be found in Online-only Tables 2–8, which corresponds to the csv files in the figshare repository. To avoid making assumptions about how the data will be used, we have included all available data from the sources. This means that in some cases the same diet samples are reported multiple times using different diet metrics. In the figshare repository, there is R v3.6.1 code (‘Preprocessing_data’) to exclude overlapping data based on diet metrics of interest: presence/absence, weight/volume, frequency of occurrence, and numerical.

A dynamic version of the North Pacific Marine Salmon Diet Database can be accessed through a GitHub repository and will be updated as more historic data are digitized and made available by the Pelagic Ecosystems Laboratory at the University of British Columbia^21^. The GitHub repository contains updated versions of the files found in the figshare repository, in addition to documentation for the North Pacific Marine Salmon Diet Database ('npmsdd_documentation’), a data entry template (‘npmsdd_data_entry_template’), and a dumped version of the MySQL relational database (‘npmsdd_MySQL_database_dump’) for users that are interested in working with the database in the relational database format.

## Data Availability

The figshare repository contains R v3.6.1 code for downstream data processing (‘Preprocessing_data’), and code to create the figures that appear in this publication (‘Publication_figures’)^22^. The GitHub repository contains the most up-to-date code for downstream data processing^21^.

## Online-only Tables

**Online-Only Table 1:** The sources included in the database.

Source_id	First Author	Year	Reference Number
1	Andrievskaya, L. D.	1966	^24^
2	Carlson, H. R.	1976	^25^
3	Chuchukalo, V. L.	1995	^26^
4	Davis, N. D.	1996	^27^
5	Davis, N. D.	1998	^28^
6	Davis, N. D.	2000	^29^
7	Dulepova, E. P.	2003	^30^
8	Fukataki, H.	1967	^31^
9	Glebov, I. I.	1998	^32^
10	Ito, J.	1964	^33^
11	Kaeriyama, M.	2000	^34^
12	Kaeriyama, M.	2004	^15^
13	Kanno, Y.	1971	^35^
14	Karpenko, V. I.	2007	^13^
15	Manzer, J. I.	1968	^36^
16	Perry, R. I.	1996	^37^
17	Tadokoro, K.	1996	^38^
18	Takeuchi, I.	1972	^39^
19	Ueno, M.	1969	^40^
20	Volkov, A. F.	1995	^41^
21	Auburn, M. E.	2000	^42^
22	Aydin, K. Y.	1998	^43^
23	Brodeur, R. D.	2007	^11^
24	Daly, E. A.	2015	^44^
25	Davis, N. D.	2003	^45^
26	Kawamura, H.	1998	^46^
27	Qin, Y.	2016	^8^
28	Starovoytov, A. N.	2007	^14^
29	Ueno, Y.	1992	^47^
30	Ueno, Y.	1992	^48^
31	Waddell, B. J.	1992	^49^
32	Andrievskaya, L. D.	1970	^50^
33	Atcheson, M. E.	2012	^51^
34	Carlson, H. R.	1996	^52^
35	Davis, N. D.	2005	^53^
36	Myers, K. W.	1996	^54^
37	Myers, K. W.	1995	^55^
38	Sturdevant, M. V.	1997	^56^
39	Walker, R. V.	1993	^57^
40	Suzuki, T.	1994	^58^
41	Shimazaki, K.	1969	^59^
42	Weitkamp, L.	2004	^60^
43	Starovoytov, A. N.	2003	^61^
44	LeBrasseur, R. J.	1966	^62^
45	LeBrasseur, R. J.	1966	^63^
46	LeBrasseur, R. J.	1966	^64^
47	Ishida, Y.	1999	^65^
48	Tamura, R.	1999	^66^
49	Seki, J.	1998	^67^
50	Suzuki, T.	1995	^68^
51	Andrievskaya, L. D.	1974	^69^
52	Andrievskaya, L. D.	1970	^70^
53	Chuchukalo, V. I.	1994	^71^
54	Gorbatenko, K. M.	1996	^72^
55	Kayev, A. M.	1993	^73^
56	Klovatch, N. V.	2002	^74^
57	Shershnev, A. P.	1982	^75^
58	Tutubalin, B. G.	1992	^76^
59	Volkov, A. F.	1996	^77^
60	Volkov, A. F.	1994	^78^
61	Fisheries Agency of Japan	1966	^79^
62	Davis, N. D.	1990	^80^

Sources are listed in order of their source_id number, which corresponds to their source_id in the database. For each source, the first author, year of publication and the reference number, which corresponds to the final ‘References’ section, are listed.

**Online-Only Table 2:** The metadata extracted for each source.

Column	Explanation
source_id	A unique number that is generated and assigned to each source (e.g., 1, 2, 3…etc.)
publication_year	Publication year in the format: YYYY
title	Title of the source
author1	First author of the source in the following format: M. C. Graham or M. Graham (first and middle names are abbreviated by initials and a period while last names are completely spelled out)
author2	Second author of the source in the following format: M. C. Graham or M. Graham (first and middle names are abbreviated by initials and a period while last names are completely spelled out)
author3	Third author of the source in the following format: M. C. Graham or M. Graham (first and middle names are abbreviated by initials and a period while last names are completely spelled out)
author4	Fourth author of the source in the following format: M. C. Graham or M. Graham (first and middle names are abbreviated by initials and a period while last names are completely spelled out)
author5	Fifth author of the source in the following format: M. C. Graham or M. Graham (first and middle names are abbreviated by initials and a period while last names are completely spelled out)
author6	Sixth author of the source in the following format: M. C. Graham or M. Graham (first and middle names are abbreviated by initials and a period while last names are completely spelled out)
author7	Seventh author of the source in the following format: M. C. Graham or M. Graham (first and middle names are abbreviated by initials and a period while last names are completely spelled out)
author8	Eighth author of the source in the following format: M. C. Graham or M. Graham (first and middle names are abbreviated by initials and a period while last names are completely spelled out)
author9	Ninth author of the source in the following format: M. C. Graham or M. Graham (first and middle names are abbreviated by initials and a period while last names are completely spelled out)
author10	Tenth author of the source in the following format: M. C. Graham or M. Graham (first and middle names are abbreviated by initials and a period while last names are completely spelled out)
url	URL associated with source, if applicable
citation	The full citation for the source
source_notes	Any additional notes about the source; for example, if the data might be overlapping with another source, this is indicated in this attribute
data_processing_notes	Notes about how the data were processed – in the lab or field, were quantities measured using scales or visually estimated, etc.
date_entered	The date the source was added to the database
entered_by	The full name of the person who entered the data (e.g., Caroline Graham)

This table corresponds to the ‘Sources’ file in the figshare repository.

**Online-Only Table 3:** The data extracted for each salmon gear type.

Column	Explanation
gear_type_predator_id	A unique number that is generated and assigned to each unique predator gear type for each source (e.g., 1, 2, 3…etc.)
gear_type	The most basic description of the type of gear given in the source (e.g., trawl, gillnet, longline)
gear_length_value	The gear length
gear_length_min	If there are a range of gear lengths, then this attribute represents the minimum length
gear_length_max	If there are a range of gear lengths, then this attribute represents the maximum length
gear_length_units	The units associated with the gear length; units are fully spelled and plural (e.g., meters instead of meter)
gear_width_value	The gear width
gear_width_min	If there are a range of gear widths, then this attribute represents the minimum width
gear_width_max	If there are a range of gear widths, then this attribute represents the maximum width
gear_width_units	The units associated with the gear width; units are fully spelled and plural (e.g., meters instead of meter)
gear_depth_value	The gear depth; if the gear is reported to be deployed at the surface then the depth value is assigned to 0
gear_depth_min	If there are a range of gear depths, then this attribute represents the minimum depth; if the gear is reported to be deployed at the surface then the depth value is assigned to 0
gear_depth_max	If there are a range of gear depths, then this attribute represents the maximum depth
gear_depth_units	The units associated with the gear depth; units are fully spelled and plural (e.g., meters instead of meter)
mesh_size_value	The gear mesh size
mesh_size_min	If there are a range of gear mesh sizes, then this attribute represents the minimum mesh size
mesh_size_max	If there are a range of gear mesh sizes, then this attribute represents the maximum mesh size
mesh_size_units	The units associated with the mesh size; units are fully spelled and plural (e.g., millimeters instead of millimeter)
fishing_depth_value	The fishing depth; if fishing is reported to be at the surface then the depth value is assigned to 0
fishing_depth_min	If there are a range of fishing depths, then this attribute represents the minimum fishing depth; if fishing is reported to be at the surface then the depth value is assigned to 0
fishing_depth_max	If there are a range of fishing depths, then this attribute represents the maximum fishing depth
fishing_depth_units	The units associated with the fishing depth; units are fully spelled and plural (e.g., meters instead of meter)
tow_speed_value	The gear tow speed
tow_speed_min	If there are a range of gear tow speeds, then this attribute represents the minimum tow speed
tow_speed_max	If there are a range of gear tow speeds, then this attribute represents the maximum tow speed
tow_speed_units	The units associated with the tow speed; units are fully spelled and plural (e.g., knots instead of knot)
duration_deployment_value	The gear duration of deployment
duration_deployment_min	If there are a range of gear durations of deployment, then this attribute represents the minimum duration of deployment
duration_deployment_max	If there are a range of gear durations of deployment, then this attribute represents the maximum duration of deployment
duration_deployment_units	The units associated with the duration of deployment; units are fully spelled and plural (e.g., minutes instead of minute)
gear_notes	Any additional comments on the gear

This table corresponds to the ‘Gear_type_predator’ file in the figshare repository.

**Online-Only Table 4:** The diet data extracted for each salmon predator.

Column	Explanation
predator_id	A unique number that is generated and assigned to each predator sample
source_id	This number corresponds with the source_id from Online-only Table 2
year_min	YYYY; if there is just one value for the year then it is found in this attribute; if there are a range of values for the year then the minimum value is found in this attribute
year_max	YYYY; if there are a range of values for the year then the maximum value is entered into this attribute
warm_cool_years	Either ‘warm’, ‘cool’ or NA; this attribute will only have a value if the samples are explicitly reported as being from a warm versus cool year(s) and can only be uniquely identified this way
odd_even_years	Either ‘odd, ‘even’ or NA; this attribute will only have a value if the samples are explicitly reported as being from an odd versus even year(s) and can only be uniquely identified this way
season_min	Either ‘spring’, ‘summer’, ‘autumn’, or ‘winter’; if there is just one value for the season then it is entered into this attribute; if there are a range of values for the season then the minimum value is entered into this attribute; this attribute will only have a value if there is no value for the month/date and the source explicitly defines the temporal sampling period by season
season_max	Either ‘spring’, ‘summer’, ‘autumn’, or ‘winter’; if there are a range of values for the season then the maximum value is entered into this attribute; this attribute will only have a value if there is no value for the month/date and the source explicitly defines the temporal sampling period by season
month_min	Month names are completely spelled out with the first letter capitalized; if there is just one value for the month then it is entered into this attribute; if there are a range of values for the month then the minimum value is found in this attribute
month_max	Month names are completely spelled out with the first letter capitalized; if there are a range of values for the month then the maximum value is found in this attribute
date_min	Expressed using year, month, and day; if there is just one value for the date then it is entered into this attribute; if there are a range of values for the date then the minimum value is found in this attribute
date_max	Expressed using year, month, and day; if there are a range of values for the date then the maximum value is found in this attribute
time_min	HH:MM:SS; if there is just one value for the time then it is found in this attribute; if there are a range of values for the time then the minimum value is found in this attribute
time_max	HH:MM:SS; if there are a range of values for the time then the maximum value is found in this attribute
lat_min	If there is just one value for the latitude then it is found in this attribute;if there are a range of values for the latitude then the minimum value is found in this attribute; values are in decimal degrees format
lat_max	If there are a range of values for the latitude then the maximum value is found in this attribute; values are in decimal degrees format
lon_min	If there is just one value for the longitude then it is entered into this attribute; if there are a range of values for the longitude then the minimum value is entered into this attribute; values are in decimal degrees format
lon_max	If there are a range of values for the longitude then the maximum value is found in this attribute; values are in decimal degrees format
predator_lowest_taxonomic_level	The lowest taxonomic level reported in the source; if a source reports to the species level then this attribute includes both the genus and species names (e.g., Oncorhynchus nerka); only scientific names are reported, not common names
predator_life_stage	Either ‘juvenile’, ‘adult’ or NA
predator_life_stage_min	If there are a mixture of juveniles and adults, then ‘juvenile’ is entered here
predator_life_stage_max	If there are a mixture of juveniles and adults, then ‘adult’ is entered here
predator_freshwater_age	An integer to indicate the number of years spent living in freshwater
predator_freshwater_age_min	If there are a mixture of freshwater ages, then this attribute represents the minimum age; an integer to indicate the number of years spent living in freshwater
predator_freshwater_age_max	If there are a mixture of freshwater ages, then this attribute represents the maximum age; an integer to indicate the number of years spent living in freshwater
predator_ocean_age	An integer to indicate the number of years spent living in the ocean
predator_ocean_age_min	If there are a mixture of ocean ages, then this attribute represents the minimum age; an integer to indicate the number of years spent living in the ocean
predator_ocean_age_max	If there are a mixture of ocean ages, then this attribute represents the maximum age; an integer to indicate the number of years spent living in the ocean
predator_maturity	Either ‘juvenile’, ‘immature’, ‘maturing’ ‘mature’, ‘kelt’ (for steelhead), or NA
predator_maturity_min	If there are a mixture of maturity levels, then the minimum maturity level is found here
predator_maturity_max	If there are a mixture of maturity levels, then the maximum maturity level is found here
predator_length_value_cm	The length of a predator in centimeters (could be either fork length or total length); only reported if there is no other way to determine life stage, or if length or weight categories are the only way to uniquely identify samples of diet data from a source
predator_length_min_cm	The minimum length of a predator in centimeters (could be either fork length or total length) if there are a range of sizes; only reported if there is no other way to determine life stage, or if length or weight categories are the only way to uniquely identify samples of diet data from a source
predator_length_max_cm	The maximum length of a predator in centimeters (could be either fork length or total length) if there are a range of sizes; only reported if there is no other way to determine life stage, or if length or weight categories are the only way to uniquely identify samples of diet data from a source
predator_weight_value_g	The weight of a predator in grams; only reported if there is no other way to determine life stage, or if length or weight categories are the only way to uniquely identify samples of diet data from a source
predator_weight_min_g	The minimum weight of a predator in grams if there are a range of sizes; only reported if there is no other way to determine life stage, or if length or weight categories are the only way to uniquely identify samples of diet data from a source
predator_weight_max_g	The maximum weight of a predator in grams if there are a range of sizes; only reported if there is no other way to determine life stage, or if length or weight categories are the only way to uniquely identify samples of diet data from a source
predator_subsample_id	A unique number that is generated and assigned to predator samples if a source reports the diets of individual predators with no unique identifiers; values are assigned for each source starting from 1 and increasing by a value of 1 each time (e.g., 1,2,3…); if unique subsample_ids are not required then the default value is 0
predator_sex	Either ‘male’, ‘female’ or ‘unspecified’
hatchery_wild	Either ‘hatchery’, ‘wild’ or ‘unspecified’
predator_replicates	The total number of predator replicates per sample
predator_notes	Any additional comments on the predator
gear_type_predator_id1	This id number corresponds with the gear_type_predator_id from Online-only Table 3
gear_type_predator_id2	This id number corresponds with the gear_type_predator_id from Online-only Table 3; this attribute is required if there are at least 2 types of gear used to sample predators
gear_type_predator_id3	This id number corresponds with the gear_type_predator_id from Online-only Table 3; this attribute is required if there are at least 3 types of gear used to sample predators
gear_type_predator_id4	This id number corresponds with the gear_type_predator_id from Online-only Table 3; this attribute is required if there are at least 4 types of gear used to sample predators
gear_type_predator_id5	This id number corresponds with the gear_type_predator_id from Online-only Table 3; this attribute is required if there are at least 5 types of gear used to sample predators
type_diet_data	The diet metric reported in the source (e.g., percent weight of prey, index of relative importance)
diet_data_units	The diet data units (e.g., percent); if the diet data are reported as a number then the units are left as blank
formula	The formula for the diet metric, if applicable; this is for metrics such as the index of relative importance or the stomach content index because they may be calculated differently in different sources
prey_lowest_taxonomic_level	The lowest taxonomic level reported in the source; if a source reports to the species level then this attribute should include both the genus and species names (e.g., Calanus pacificus); in some cases the lowest taxonomic level is not a scientific name – like ‘gelatinous’ or ‘zooplankton_collective’ or ‘miscellaneous’
prey_kingdom	The kingdom based on the lowest taxonomic level
prey_phylum	The phylum based on the lowest taxonomic level
prey_class	The class based on the lowest taxonomic level
prey_order	The order based on the lowest taxonomic level
prey_family	The family based on the lowest taxonomic level
prey_genus	The genus based on the lowest taxonomic level
prey_species	The species based on the lowest taxonomic level
value_diet_data	Diet data values for the specific prey item and the sample
prey_sex	Either ‘male’, ‘female’ or ‘unspecified’
prey_life_stage	The prey life stage
prey_life_stage_min	If there are a mixture of life stages, then this attribute represents the minimum life stage
prey_life_stage_max	If there are a mixture of life stages, then this attribute represents the maximum life stage
prey_length_value_mm	The length of prey in millimeters; only reported if there is no other way to determine life stage, or if length or weight categories are the only way to uniquely identify samples of prey data from a source
prey_length_min_mm	The minimum length of prey in millimeters if there are a range of sizes; only reported if there is no other way to determine life stage, or if length or weight categories are the only way to uniquely identify samples of prey data from a source
prey_length_max_mm	The maximum length of prey in millimeters if there are a range of sizes; only reported if there is no other way to determine life stage, or if length or weight categories are the only way to uniquely identify samples of prey data from a source
prey_weight_value_mg	The weight of prey in milligrams; only reported if there is no other way to determine life stage, or if length or weight categories are the only way to uniquely identify samples of prey data from a source
prey_weight_min_mg	The minimum weight of prey in milligrams if there are a range of sizes; only reported if there is no other way to determine life stage, or if length or weight categories are the only way to uniquely identify samples of prey data from a source
prey_weight_max_mg	The maximum weight of prey in milligrams if there are a range of sizes; only reported if there is no other way to determine life stage, or if length or weight categories are the only way to uniquely identify samples of prey data from a source
prey_notes	Any additional comments on prey

This table corresponds to the ‘Diet_data’ file in the figshare repository.

**Online-Only Table 5:** The associated salmon biological data for each salmon sample.

Column	Explanation
predator_id	A unique number that is generated and assigned to each predator sample
source_id	This number corresponds with the source_id from Online-only Table 2
year_min	YYYY; if there is just one value for the year then it is found in this attribute; if there are a range of values for the year then the minimum value is found in this attribute
year_max	YYYY; if there are a range of values for the year then the maximum value is entered into this attribute
warm_cool_years	Either ‘warm’, ‘cool’ or NA; this attribute will only have a value if the samples are explicitly reported as being from a warm versus cool year(s) and can only be uniquely identified this way
odd_even_years	Either ‘odd, ‘even’ or NA; this attribute will only have a value if the samples are explicitly reported as being from an odd versus even year(s) and can only be uniquely identified this way
season_min	Either ‘spring’, ‘summer’, ‘autumn’, or ‘winter’; if there is just one value for the season then it is entered into this attribute; If there are a range of values for the season then the minimum value is entered into this attribute; this attribute will only have a value if there is no value for the month/date and the source explicitly defines the temporal sampling period by season
season_max	Either ‘spring’, ‘summer’, ‘autumn’, or ‘winter’; if there are a range of values for the season then the maximum value is entered into this attribute; this attribute will only have a value if there is no value for the month/date and the source explicitly defines the temporal sampling period by season
month_min	Month names are completely spelled out with the first letter capitalized; if there is just one value for the month then it is entered into this attribute; If there are a range of values for the month then the minimum value is found in this attribute
month_max	Month names are completely spelled out with the first letter capitalized; if there are a range of values for the month then the maximum value is found in this attribute
date_min	Expressed using year, month, and day; if there is just one value for the date then it is entered into this attribute; if there are a range of values for the date then the minimum value is found in this attribute
date_max	Expressed using year, month, and day; if there are a range of values for the date then the maximum value is found in this attribute
time_min	HH:MM:SS; if there is just one value for the time then it is found in this attribute; if there are a range of values for the time then the minimum value is found in this attribute
time_max	HH:MM:SS; if there are a range of values for the time then the maximum value is found in this attribute
lat_min	If there is just one value for the latitude then it is found in this attribute; if there are a range of values for the latitude then the minimum value is found in this attribute; values are in decimal degrees format
lat_max	If there are a range of values for the latitude then the maximum value is found in this attribute; values are in decimal degrees format
lon_min	If there is just one value for the longitude then it is entered into this attribute; if there are a range of values for the longitude then the minimum value is entered into this attribute; values are in decimal degrees format
lon_max	If there are a range of values for the longitude then the maximum value is found in this attribute; values are in decimal degrees format
predator_lowest_taxonomic_level	The lowest taxonomic level reported in the source; if a source reports to the species level then this attribute includes both the genus and species names (e.g., Oncorhynchus nerka); only scientific names are reported, not common names
predator_life_stage	Either ‘juvenile’, ‘adult’ or NA
predator_life_stage_min	If there are a mixture of juveniles and adults, then ‘juvenile’ is entered here
predator_life_stage_max	If there are a mixture of juveniles and adults, then ‘adult’ is entered here
predator_freshwater_age	An integer to indicate the number of years spent living in freshwater
predator_freshwater_age_min	If there are a mixture of freshwater ages, then this attribute represents the minimum age; an integer to indicate the number of years spent living in freshwater
predator_freshwater_age_max	If there are a mixture of freshwater ages, then this attribute represents the maximum age; an integer to indicate the number of years spent living in freshwater
predator_ocean_age	An integer to indicate the number of years spent living in the ocean
predator_ocean_age_min	If there are a mixture of ocean ages, then this attribute represents the minimum age; an integer to indicate the number of years spent living in the ocean
predator_ocean_age_max	If there are a mixture of ocean ages, then this attribute represents the maximum age; an integer to indicate the number of years spent living in the ocean
predator_maturity	Either ‘juvenile’, ‘immature’, ‘maturing’ ‘mature’, ‘kelt’ (for steelhead), or NA
predator_maturity_min	If there are a mixture of maturity levels, then the minimum maturity level is found here
predator_maturity_max	If there are a mixture of maturity levels, then the maximum maturity level is found here
predator_length_value_cm	The length of a predator in centimeters (could be either fork length or total length); only reported if there is no other way to determine life stage, or if length or weight categories are the only way to uniquely identify samples of diet data from a source
predator_length_min_cm	The minimum length of a predator in centimeters (could be either fork length or total length) if there are a range of sizes; only reported if there is no other way to determine life stage, or if length or weight categories are the only way to uniquely identify samples of diet data from a source
predator_length_max_cm	The maximum length of a predator in centimeters (could be either fork length or total length) if there are a range of sizes; only reported if there is no other way to determine life stage, or if length or weight categories are the only way to uniquely identify samples of diet data from a source
predator_weight_value_g	The weight of a predator in grams; only reported if there is no other way to determine life stage, or if length or weight categories are the only way to uniquely identify samples of diet data from a source
predator_weight_min_g	The minimum weight of a predator in grams if there are a range of sizes; only reported if there is no other way to determine life stage, or if length or weight categories are the only way to uniquely identify samples of diet data from a source
predator_weight_max_g	The maximum weight of a predator in grams if there are a range of sizes; only reported if there is no other way to determine life stage, or if length or weight categories are the only way to uniquely identify samples of diet data from a source
predator_subsample_id	A unique number that is generated and assigned to predator samples if a source reports the diets of individual predators with no unique identifiers; values are assigned for each source starting from 1 and increasing by a value of 1 each time (e.g., 1,2,3…); if unique subsample_ids are not required then the default value is 0
predator_sex	Either ‘male’, ‘female’ or ‘unspecified’
hatchery_wild	Either ‘hatchery’, ‘wild’ or ‘unspecified’
predator_replicates	The total number of predator replicates per sample
predator_notes	Any additional comments on the predator
gear_type_predator_id1	This id number corresponds with the gear_type_predator_id from Online-only Table 3
gear_type_predator_id2	This id number corresponds with the gear_type_predator_id from Online-only Table 3; this attribute is required if there are at least 2 types of gear used to sample predators
gear_type_predator_id3	This id number corresponds with the gear_type_predator_id from Online-only Table 3; this attribute is required if there are at least 3 types of gear used to sample predators
gear_type_predator_id4	This id number corresponds with the gear_type_predator_id from Online-only Table 3; this attribute is required if there are at least 4 types of gear used to sample predators
gear_type_predator_id5	This id number corresponds with the gear_type_predator_id from Online-only Table 3; this attribute is required if there are at least 5 types of gear used to sample predators
biological_parameter	The predator biological parameter reported in the source (e.g., total length, fork length, body weight)
predator_bio_notes	Any additional comments on the predator biological parameters
value	The biological parameter value
mean	The biological parameter mean
error	The error associated with the biological parameter mean
min	If there are a range of values for the biological parameter this attribute represents the minimum value
max	If there are a range of values for the biological parameter this attribute represents the maximum value
units	The units associated with the biological parameter; units are fully spelled and plural (e.g., centimeters instead of centimeter)

This table corresponds to the ‘Predator_biological_data’ file in the figshare repository.

**Online-Only Table 6:** The data extracted for each prey gear type.

Column	Explanation
gear_type_prey_id	A unique number that is generated and assigned to each unique prey gear type for each source (e.g., 1, 2, 3…etc.)
gear_type	The most basic description of the type of gear given in the source (e.g., bongo net); if the prey is a diet item, then the gear_type is ‘predator’ to indicate that it was not collected from the environment but instead in a salmon stomach
gear_length_value	The gear length
gear_length_min	If there are a range of gear lengths, then this attribute represents the minimum length
gear_length_max	If there are a range of gear lengths, then this attribute represents the maximum length
gear_length_units	The units associated with the gear length; units are fully spelled and plural (e.g., meters instead of meter)
gear_width_value	The gear width
gear_width_min	If there are a range of gear widths, then this attribute represents the minimum width
gear_width_max	If there are a range of gear widths, then this attribute represents the maximum width
gear_width_units	The units associated with the gear width; units are fully spelled and plural (e.g., meters instead of meter)
gear_depth_value	The gear depth; if gear is reported to be deployed at the surface then the depth value is assigned to 0
gear_depth_min	If there are a range of gear depths, then this attribute represents the minimum depth; if gear is reported to be deployed at the surface then the depth value is assigned to 0
gear_depth_max	If there are a range of gear depths, then this attribute represents the maximum depth
gear_depth_units	The units associated with the gear depth; units are fully spelled and plural (e.g., meters instead of meter)
mesh_size_value	The gear mesh size
mesh_size_min	If there are a range of gear mesh sizes, then this attribute represents the minimum mesh size
mesh_size_max	If there are a range of gear mesh sizes, then this attribute represents the maximum mesh size
mesh_size_units	The units associated with the mesh size; units are fully spelled and plural (e.g., millimeters instead of millimeter)
fishing_depth_value	The fishing depth; if fishing is reported to be at the surface then the depth value is assigned to 0
fishing_depth_min	If there are a range of fishing depths, then this attribute represents the minimum fishing depth; if fishing is reported to be at the surface then the depth value is assigned to 0
fishing_depth_max	If there are a range of fishing depths, then this attribute represents the maximum fishing depth
fishing_depth_units	The units associated with the fishing depth; units are fully spelled and plural (e.g., meters instead of meter)
tow_speed_value	The gear tow speed
tow_speed_min	If there are a range of gear tow speeds, then this attribute represents the minimum tow speed
tow_speed_max	If there are a range of gear tow speeds, then this attribute represents the maximum tow speed
tow_speed_units	The units associated with the tow speed; units are fully spelled and plural (e.g., knots instead of knot)
duration_deployment_value	The gear duration of deployment
duration_deployment_min	If there are a range of gear durations of deployment, then this attribute represents the minimum duration of deployment
duration_deployment_max	If there are a range of gear durations of deployment, then this attribute represents the maximum duration of deployment
duration_deployment_units	The units associated with the duration of deployment; units are fully spelled and plural (e.g., minutes instead of minute)
gear_notes	Any additional comments on the gear

This table corresponds to the ‘Gear_type_prey’ file in the figshare repository.

**Online-Only Table 7:** The associated prey biological data for each prey sample.

Column	Explanation
prey_id	A unique number that is generated and assigned to each prey sample
predator_id	A unique number that is generated and assigned to each predator sample
source_id	This number corresponds with the source_id from Online-only Table 2
year_min	YYYY; if there is just one value for the year then it is found in this attribute; if there are a range of values for the year then the minimum value is found in this attribute
year_max	YYYY; if there are a range of values for the year then the maximum value is entered into this attribute
warm_cool_years	Either ‘warm’, ‘cool’ or NA; this attribute will only have a value if the samples are explicitly reported as being from a warm versus cool year(s) and can only be uniquely identified this way
odd_even_years	Either ‘odd, ‘even’ or NA; this attribute will only have a value if the samples are explicitly reported as being from an odd versus even year(s) and can only be uniquely identified this way
season_min	Either ‘spring’, ‘summer’, ‘autumn’, or ‘winter’; if there is just one value for the season then it is entered into this attribute; If there are a range of values for the season then the minimum value is entered into this attribute; this attribute will only have a value if there is no value for the month/date and the source explicitly defines the temporal sampling period by season
season_max	Either ‘spring’, ‘summer’, ‘autumn’, or ‘winter’; if there are a range of values for the season then the maximum value is entered into this attribute; this attribute will only have a value if there is no value for the month/date and the source explicitly defines the temporal sampling period by season
month_min	Month names are completely spelled out with the first letter capitalized; if there is just one value for the month then it is entered into this attribute; If there are a range of values for the month then the minimum value is found in this attribute
month_max	Month names are completely spelled out with the first letter capitalized; if there are a range of values for the month then the maximum value is found in this attribute
date_min	Expressed using year, month, and day; if there is just one value for the date then it is entered into this attribute; if there are a range of values for the date then the minimum value is found in this attribute
date_max	Expressed using year, month, and day; if there are a range of values for the date then the maximum value is found in this attribute
time_min	HH:MM:SS; if there is just one value for the time then it is found in this attribute; if there are a range of values for the time then the minimum value is found in this attribute
time_max	HH:MM:SS; if there are a range of values for the time then the maximum value is found in this attribute
lat_min	If there is just one value for the latitude then it is found in this attribute; if there are a range of values for the latitude then the minimum value is found in this attribute; values are in decimal degrees format
lat_max	If there are a range of values for the latitude then the maximum value is found in this attribute; values are in decimal degrees format
lon_min	If there is just one value for the longitude then it is entered into this attribute; if there are a range of values for the longitude then the minimum value is entered into this attribute; values are in decimal degrees format
lon_max	If there are a range of values for the longitude then the maximum value is found in this attribute; values are in decimal degrees format
predator_lowest_taxonomic_level	The lowest taxonomic level reported in the source; if a source reports to the species level then this attribute includes both the genus and species names (e.g., Oncorhynchus nerka); only scientific names are reported, not common names
predator_life_stage	Either ‘juvenile’, ‘adult’ or NA
predator_life_stage_min	If there are a mixture of juveniles and adults, then ‘juvenile’ is entered here
predator_life_stage_max	If there are a mixture of juveniles and adults, then ‘adult’ is entered here
predator_freshwater_age	An integer to indicate the number of years spent living in freshwater
predator_freshwater_age_min	If there are a mixture of freshwater ages, then this attribute represents the minimum age; an integer to indicate the number of years spent living in freshwater
predator_freshwater_age_max	If there are a mixture of freshwater ages, then this attribute represents the maximum age; an integer to indicate the number of years spent living in freshwater
predator_ocean_age	An integer to indicate the number of years spent living in the ocean
predator_ocean_age_min	If there are a mixture of ocean ages, then this attribute represents the minimum age; an integer to indicate the number of years spent living in the ocean
predator_ocean_age_max	If there are a mixture of ocean ages, then this attribute represents the maximum age; an integer to indicate the number of years spent living in the ocean
predator_maturity	Either ‘juvenile’, ‘immature’, ‘maturing’ ‘mature’, ‘kelt’ (for steelhead), or NA
predator_maturity_min	If there are a mixture of maturity levels, then the minimum maturity level is found here
predator_maturity_max	If there are a mixture of maturity levels, then the maximum maturity level is found here
predator_length_value_cm	The length of a predator in centimeters (could be either fork length or total length); only reported if there is no other way to determine life stage, or if length or weight categories are the only way to uniquely identify samples of diet data from a source
predator_length_min_cm	The minimum length of a predator in centimeters (could be either fork length or total length) if there are a range of sizes; only reported if there is no other way to determine life stage, or if length or weight categories are the only way to uniquely identify samples of diet data from a source
predator_length_max_cm	The maximum length of a predator in centimeters (could be either fork length or total length) if there are a range of sizes; only reported if there is no other way to determine life stage, or if length or weight categories are the only way to uniquely identify samples of diet data from a source
predator_weight_value_g	The weight of a predator in grams; only reported if there is no other way to determine life stage, or if length or weight categories are the only way to uniquely identify samples of diet data from a source
predator_weight_min_g	The minimum weight of a predator in grams if there are a range of sizes; only reported if there is no other way to determine life stage, or if length or weight categories are the only way to uniquely identify samples of diet data from a source
predator_weight_max_g	The maximum weight of a predator in grams if there are a range of sizes; only reported if there is no other way to determine life stage, or if length or weight categories are the only way to uniquely identify samples of diet data from a source
predator_subsample_id	A unique number that is generated and assigned to predator samples if a source reports the diets of individual predators with no unique identifiers; values are assigned for each source starting from 1 and increasing by a value of 1 each time (e.g., 1,2,3…); if unique subsample_ids are not required then the default value is 0
predator_sex	Either ‘male’, ‘female’ or ‘unspecified’
hatchery_wild	Either ‘hatchery’, ‘wild’ or ‘unspecified’
predator_replicates	The total number of predator replicates per sample
predator_notes	Any additional comments on the predator
gear_type_predator_id1	This id number corresponds with the gear_type_predator_id from Online-only Table 3
gear_type_predator_id2	This id number corresponds with the gear_type_predator_id from Online-only Table 3; this attribute is required if there are at least 2 types of gear used to sample predators
gear_type_predator_id3	This id number corresponds with the gear_type_predator_id from Online-only Table 3; this attribute is required if there are at least 3 types of gear used to sample predators
gear_type_predator_id4	This id number corresponds with the gear_type_predator_id from Online-only Table 3; this attribute is required if there are at least 4 types of gear used to sample predators
gear_type_predator_id5	This id number corresponds with the gear_type_predator_id from Online-only Table 3; this attribute is required if there are at least 5 types of gear used to sample predators
prey_lowest_taxonomic_level	The lowest taxonomic level reported in the source; if a source reports to the species level then this attribute includes both the genus and species names (e.g., Calanus pacificus); in some cases the lowest taxonomic level is not a scientific name – like ‘gelatinous’ or ‘zooplankton_collective’ or ‘miscellaneous’
prey_kingdom	The kingdom based on the lowest taxonomic level
prey_phylum	The phylum based on the lowest taxonomic level
prey_class	The class based on the lowest taxonomic level
prey_order	The order based on the lowest taxonomic level
prey_family	The family based on the lowest taxonomic level
prey_genus	The genus based on the lowest taxonomic level
prey_species	The species based on the lowest taxonomic level
prey_life_stage	The prey life stage
prey_life_stage_min	If there are a mixture of life stages, then this attribute represents the minimum life stage
prey_life_stage_max	If there are a mixture of life stages, then this attribute represents the maximum life stage
prey_length_value_mm	The length of prey in millimeters; only reported if there is no other way to determine life stage, or if length or weight categories are the only way to uniquely identify samples of prey data from a source
prey_length_min_mm	The minimum length of prey in millimeters if there are a range of sizes; only reported if there is no other way to determine life stage, or if length or weight categories are the only way to uniquely identify samples of prey data from a source
prey_length_max_mm	The maximum length of prey in millimeters if there are a range of sizes; only reported if there is no other way to determine life stage, or if length or weight categories are the only way to uniquely identify samples of prey data from a source
prey_weight_value_mg	The weight of prey in milligrams; only reported if there is no other way to determine life stage, or if length or weight categories are the only way to uniquely identify samples of prey data from a source
prey_weight_min_mg	The minimum weight of prey in milligrams if there are a range of sizes; only reported if there is no other way to determine life stage, or if length or weight categories are the only way to uniquely identify samples of prey data from a source
prey_weight_max_mg	The maximum weight of prey in milligrams if there are a range of sizes; only reported if there is no other way to determine life stage, or if length or weight categories are the only way to uniquely identify samples of prey data from a source
prey_sex	Either ‘male’, ‘female’ or ‘unspecified’
prey_subsample_id	A unique number that is generated and assigned to prey samples if a source reports the biological parameters of individual prey with no unique identifiers; values are assigned for each source starting from 1 and increasing by a value of 1 each time (e.g., 1,2,3…)
prey_replicates	The total number of prey replicates per sample
prey_notes	Any additional comments on the prey
gear_type_prey_id1	This id number corresponds with the gear_type_prey_id from Online-only Table 6; if the prey is part of a diet data sample then the id should be 1 which corresponds to the ‘predator’ gear type (i.e. sample came from a predator stomach)
gear_type_prey_id2	This id number corresponds with the gear_type_prey_id from Online-only Table 6; this attribute is required if there are at least 2 types of gear used to sample predators
gear_type_prey_id3	This id number corresponds with the gear_type_prey_id from Online-only Table 6; this attribute is required if there are at least 3 types of gear used to sample predators
gear_type_prey_id4	This id number corresponds with the gear_type_prey_id from Online-only Table 6; this attribute is required if there are at least 4 types of gear used to sample predators
gear_type_prey_id5	This id number corresponds with the gear_type_prey_id from Online-only Table 6; this attribute is required if there are at least 5 types of gear used to sample predators
biological_parameter	The prey biological parameter reported in the source (e.g., body length, body weight)
prey_bio_notes	Any additional comments on the prey biological parameters
value	The biological parameter value
mean	The biological parameter mean
error	The error associated with the biological parameter mean
min	If there are a range of values for the biological parameter this attribute represents the minimum value
max	If there are a range of values for the biological parameter this attribute represents the maximum value
units	The units associated with the biological parameter; units are fully spelled and plural (e.g., centimeters instead of centimeter)

This data could come from diet samples or from environmental samples of potential prey items. This table corresponds to the ‘Prey_biological_data’ file in the figshare repository.

**Online-Only Table 8:** The associated environmental data for each salmon or prey sample.

Column	Explanation
environmental_data_id	A unique number that is generated and assigned to each environmental data point
source_id	This number corresponds with the source_id from Online-only Table 2
year_min	YYYY; if there is just one value for the year then it is found in this attribute; if there are a range of values for the year then the minimum value is found in this attribute
year_max	YYYY; if there are a range of values for the year then the maximum value is entered into this attribute
warm_cool_years	Either ‘warm’, ‘cool’ or NA; this attribute will only have a value if the samples are explicitly reported as being from a warm versus cool year(s) and can only be uniquely identified this way
odd_even_years	Either ‘odd, ‘even’ or NA; this attribute will only have a value if the samples are explicitly reported as being from an odd versus even year(s) and can only be uniquely identified this way
season_min	Either ‘spring’, ‘summer’, ‘autumn’, or ‘winter’; if there is just one value for the season then it is entered into this attribute; If there are a range of values for the season then the minimum value is entered into this attribute; this attribute will only have a value if there is no value for the month/date and the source explicitly defines the temporal sampling period by season
season_max	Either ‘spring’, ‘summer’, ‘autumn’, or ‘winter’; if there are a range of values for the season then the maximum value is entered into this attribute; this attribute will only have a value if there is no value for the month/date and the source explicitly defines the temporal sampling period by season
month_min	Month names are completely spelled out with the first letter capitalized; if there is just one value for the month then it is entered into this attribute; If there are a range of values for the month then the minimum value is found in this attribute
month_max	Month names are completely spelled out with the first letter capitalized; if there are a range of values for the month then the maximum value is found in this attribute
date_min	Expressed using year, month, and day; if there is just one value for the date then it is entered into this attribute; if there are a range of values for the date then the minimum value is found in this attribute
date_max	Expressed using year, month, and day; if there are a range of values for the date then the maximum value is found in this attribute
time_min	HH:MM:SS; if there is just one value for the time then it is found in this attribute; if there are a range of values for the time then the minimum value is found in this attribute
time_max	HH:MM:SS; if there are a range of values for the time then the maximum value is found in this attribute
lat_min	If there is just one value for the latitude then it is found in this attribute; if there are a range of values for the latitude then the minimum value is found in this attribute; values are in decimal degrees format
lat_max	If there are a range of values for the latitude then the maximum value is found in this attribute; values are in decimal degrees format
lon_min	If there is just one value for the longitude then it is entered into this attribute; if there are a range of values for the longitude then the minimum value is entered into this attribute; values are in decimal degrees format
lon_max	If there are a range of values for the longitude then the maximum value is found in this attribute; values are in decimal degrees format
environmental_data_type	The environmental data type reported in the source (e.g., temperature, salinity)
measurement_depth	The depth associated with the environmental parameter measurement; if the measurement in reported to be at the surface (e.g., sea surface temperature) then the measurement depth value is assigned to 0
depth_units	The units associated with the measurement depth; units are fully spelled and plural (e.g., meters instead of meter)
environmental_subsample_id	A unique number that is generated and assigned to environmental samples if there are no other unique identifiers; values are assigned for each source starting from 1 and increasing by a value of 1 each time (e.g., 1,2,3…)
environmental_notes	Any additional comments on the environmental parameter measurement
value	The value of the environmental parameter
mean	The mean of the environmental parameters
error	The error associated with the environmental parameter mean
min	If there are a range of values for the environmental parameter this attribute represents the minimum value
max	If there are a range of values for the environmental parameter this attribute represents the maximum value
environmental_units	The units associated with the environmental parameter; units are fully spelled and plural (e.g., micrograms per liter instead of microgram per liter)

This table corresponds to the ‘Environmental_data’ file in the figshare repository.
